# Amendment of International Funding Resource Approaches Required to Control HIV

**Published:** 2020-04

**Authors:** Mojtaba MEHTARPOUR, Hossein BANNAZADEH BAGHI, Hamed EBRAHIMZADEH LEYLABADLO

**Affiliations:** 1. Department of Health Management and Economics, School of Public Health, Tehran University of Medical Sciences, Tehran, Iran; 2. Immunology Research Center, Tabriz University of Medical Sciences, Tabriz, Iran; 3. Infectious and Tropical Diseases Research Center, Tabriz University of Medical Sciences, Tabriz, Iran; 4. Student Research Committee, Tabriz University of Medical Sciences, Tabriz, Iran

## Dear Editor-in-Chief

International aid, as a financing mechanism, has played a major role in promoting public health, especially in poorer countries. WHO recommends the development of partnerships and international assistance as one of the most effective solutions for generating sufficient resources (1). The financial assistance for HIV has increased by more than $6 billion between 2000 and 2010, which is more than twice as high in other health sectors. Moreover, a reduction in mortality caused by the disease has been proven due to aid and present antiviral treatments (2). The United States is the largest contributor to the AIDS epidemic control in the world. The IHME dataset shows that in 2016, the United States has contributed $6.7 billion to the provision of the resources for HIV AIDS which is significantly different compared to other countries (3). Pepfar, as one of the nation’s largest commitment to combat specific diseases in more than 50 countries throughout history, has taken successful steps to control and eliminate the AIDS epidemic as a public health problem. Overall, 15.2 million Voluntary male circumcision to prevent infections, 2.2 million AIDS-free births (the newborns could potentially have been infected with AIDS if they were not supported by the program), training 250000 health workers and supporting antiretroviral therapy for 13.3 million people by 2017 and 85.5 million HIV testing in 2017 are examples of the achievements of this program(4).

However, a worrying fact remains: global health history shows that budgets and priorities have not always been sustained. In 2008, the Obama administration and health and development experts opposed the rise of US investment by thwarting the Pepfar plan. The current US government has even proposed a reduction in funding for foreign aid by up to one third, which could affect the services delivered by Pepfar in areas like prevention, care and research, the Global Fund to Fight Malaria, HIV and Tuberculosis, the National Institutes of Health, the Center for Disease Control And prevention, and US agency for international development (5). The new US government is determined to reduce foreign aid. The “America First” agenda reduced the budget for public health and foreign assistance on the agenda, creating concerns for global health. The release of the 2018 US Proposed Budget, which was expected according to Trump’s promises in his campaign, has held widespread changes to the public health budget. Trump’s insistence on a sharp decline in global health spending makes it inevitable that many programs will shut down. In the proposed budget, the AIDS budget will reach zero, US contributions to the Global Fund to Fight AIDS, Tuberculosis and Malaria ($ 225m) has been reduced dramatically and support for international organizations will be reduced by 44% (6).

Reducing international aid to the HIV program in low-income countries has blocked the way for making progress in the long route to control and eliminate the global HIV epidemic (5). The health sector has been dependent on these external sources in many recipient countries, while Experiences suggest that a recession can interrupt or reduce foreign aid and unpredictable and unsustainable aid makes planning very difficult for the recipient countries (1). Time has come for low-income countries to bear the weight of their own problems, so actions are needed to be taken to cut off the dependency of recipient countries. To reach the end of the AIDS epidemic by 2030 (according to the SDG goals), developing a capacity to manage their future as the heart of the ACRA guidelines is the best way to cut off the dependency of recipient countries. In this regard, countries must, in addition to receiving foreign assistance as part of the health sector resources, simultaneously pursue some actions such as increasing the effectiveness of tax collection, prioritizing government funding for more resources and using initiatives to raise additional resources.
